# Neuroprotection by Cerebrolysin and Citicoline Through the Upregulation of Brain-Derived Neurotrophic Factor (BDNF) Expression in the Affected Neural Cells: A Preliminary Clue Obtained Through an In Vitro Study

**DOI:** 10.7759/cureus.54665

**Published:** 2024-02-21

**Authors:** Anandan P, Santhanam Rengarajan, Sankar Venkatachalam, Sasikumar Pattabi, Sumathi Jones, Prabhu K, Vani Krishna, Krishna Prasanth

**Affiliations:** 1 Department of General Medicine, Sree Balaji Medical College and Hospital, Bharath Institute of Higher Education and Research, Chennai, IND; 2 Department of Neurosurgery, Sree Balaji Medical College and Hospital, Bharath Institute of Higher Education and Research, Chennai, IND; 3 Department of Anatomy, Dr. A.L.M. PG Institute of Basic Medical Sciences, University of Madras, Chennai, IND; 4 Department of Surgery, Sree Balaji Medical College and Hospital, Bharath Institute of Higher Education and Research, Chennai, IND; 5 Department of Pharmacology and Therapeutics, Sree Balaji Dental College and Hospital, Bharath Institute of Higher Education and Research, Chennai, IND; 6 Department of Anatomy, Sree Balaji Medical College and Hospital, Bharath Institute of Higher Education and Research, Chennai, IND; 7 Department of Community Medicine, Sree Balaji Medical College and Hospital, Bharath Institute of Higher Education and Research, Chennai, IND

**Keywords:** neuroprotective agents, bdnf, ischemic neuronal injury, citicoline, cerebrolysin

## Abstract

Objectives: Citicoline and cerebrolysin are two unique yet contentious medications because of inconsistencies in efficacy as well as the mystery surrounding their mode of action. The current study aimed to re-validate the neuroprotective benefits of these medications and investigate the possible molecular mechanism.

Methods: Neuro-2A cells were exposed to tert-butyl hydroperoxide, a consistent in vitro model of neuronal damage caused by oxidative stress. The 3-(4,5-dimethylthiazol-2-yl)-2,5-diphenyltetrazolium bromide (MTT) assay, acridine orange/ethidium bromide (AO-EtBr) staining, and phase-view examinations were utilized to evaluate cell survival and cytotoxicity. Real-time reverse transcription-polymerase chain reaction (RT-PCR)-based gene expression studies were conducted.

Key finding: Observations revealed that these two medications had modest but considerable neuroprotective effects. While the majority of the genes' expressions remained unchanged, cerebrolysin upregulated Neuregulin 1, and both upregulated brain-derived neurotrophic factor (BDNF) expression.

Conclusion: The findings of the current study may be the first to suggest that citicoline and cerebrolysin may increase host cells' defense mechanisms (secretion neurotrophic factors) rather than carrying nutrients for cell survival. Because of its simplicity, the current study can readily be repeated to learn more about these two disputed medications for treating ischemic stroke.

## Introduction

The effects of ischemia-induced pathological alterations in the central nervous system are widely documented. During the ischemia and reperfusion processes, free radicals are released, causing oxidative stress in neurons [1]. Despite numerous stroke therapy options, therapeutic care for cerebral ischemia is frequently limited to thrombolytic and anticoagulant medications [2,3].

Cerebrolysin and citicoline are two medications used to treat several neurological disorders, including stroke. Cerebrolysin, a peptidergic medication, is composed of 75% free amino acids and 25% low-molecular-weight peptides [4]. It was discovered to have positive benefits in both in vitro and in vivo experiments [5]. However, the likely mechanism underlying the purported positive effects remains unknown, and meta-analyses have not supported the use of cerebrolysin for stroke [6]. Similarly, citicoline (cytidine 5'-diphosphocholine) is a precursor of phosphatidylcholine, a component of the cell membrane [7]. Its ability to cure neurological problems is unclear. While citicoline was reported to be effective [8,9], some reports questioned its efficacy [10].

Despite these discrepancies, physicians frequently use these two drugs. In these conditions, the goal was to re-explore the potential of these two medications through experimental investigations. To achieve this goal, in the first phase, an in vitro study (cell culture method) was carried out to analyze the neuroprotective effect of these drugs as well as determine the likely mechanism in the event of any beneficial effects produced by them. The present study will provide an in vitro architecture with a consistent and reproducible platform for testing these medications.

## Materials and methods

Drugs were examined for their efficiency in recovering cultured neurons during the "reperfusion period" after oxidative stress. If neuroprotection occurred, it was assessed using the 3-(4,5-dimethylthiazol-2-yl)-2,5-diphenyltetrazolium bromide (MTT) cytotoxicity assay. In addition to the phase-view analysis of the cells, they were stained with acridine orange/ethidium bromide (AO-EtBr) to detect cell death.

Cell culture conditions

It was decided to employ a well-known cell line that is available worldwide. The study used Neuro-2A (mouse neuroblastoma cell line). This cell line was obtained from the National Centre for Cell Science (NCCS), University of Pune Campus in Maharashtra, India. The cells were grown in Dulbecco's Modified Eagle Medium (DMEM) with 4.5 g glucose/l, L-glutamine 200 mM, gentamicin 50 µg/ml, amphotericin B 2.5 µg/ml, and 10% fetal bovine serum (FBS). All of the reagents were obtained from HiMedia (Mumbai, India). Cells were cultured at 37°C in 5% CO2 and passed in a 1:2 ratio when they reached 80-90% confluence. Cells were cultured on uncoated tissue culture-grade plastic dishes, either 24-well plates or T25 flasks, as needed.

Estimation of cytotoxicity and viability

The results of the various stages of the investigation were examined by calculating the cytotoxicity of the cells and their viability. After the treatment period, 100 μl of DMEM with MTT (0.5 mg/ml) was added to each well and incubated in 5% CO2 at 37°C for three hours. The media were then withdrawn, and the cells were washed twice with phosphate-buffered saline (PBS). Living cells can convert tetrazolium into dark blue, insoluble formazan crystals. The crystals were solubilized with dimethyl sulfoxide (DMSO) and measured at 570 nm with a microplate reader. The live, healthy cells were expressed as a proportion of the control Neuro-2A cells using the following formula: the absorbance of treated cells × 100 = viability of cells in %.

The absorbance of control cells

Following phase contrast microscope observations, cells were stained with AO and EtBr to assess cell viability, as described by Mironova et al. [11]. The results were evaluated with a Nikon inverted microscope (Minato City, Tokyo, Japan) equipped with epifluorescence attachments.

Method of oxidative stress induction

Cells were grown in media supplemented with various concentrations of FBS (0%, 2%, 10%, and 20%). Both 0% and 20% serum were found to be harmful to the cells. 10% serum was beneficial to the cells and promoted growth. Because 2% serum was discovered to not affect the cells while also limiting cell proliferation, we opted to utilize it for tests because we preferred a steady population of cells without self-proliferation.

Tert-butyl hydroperoxide (tBuOOH) has been shown to cause oxidative stress in Neuro-2A cells, resulting in necrotic cell death that simulates neuronal death following an injury [12]. As a result, tBuOOH at various concentrations was employed to calculate the LD50 in cells after 24 hours. Exposure to tBuOOH at a concentration of 250 µM for 45 minutes caused 50% cell death after 24 hours after reperfusion.

Determination of the optimal dose for cerebrolysin and citicoline

Cells were seeded in media containing 2% serum and allowed to attach to the dish for 24 hours prior to drug administration. The participants were treated with various amounts of cerebrolysin (1.6, 0.8, 0.4, and 0.2 μg/ml) and citicoline (10, 1, 0.1, and 0.01 mM). Cerebrolysin at 0.2 µg/ml and citicoline at 0.1 mM dosages showed the least cytotoxicity and were employed in additional investigations.

Effects of cerebrolysin and citicoline on neurons subjected to oxidative injury

Cells were seeded in a medium with 2% serum and were allowed to attach to the dish for 24 hours. The oxidative damage was caused by exposure to 250 µM of tBuOOH for 45 minutes, as previously described (vide supra). To simulate reperfusion after injury, the medium containing tBuOOH was replaced with either cerebrolysin (0.2 µg/ml) or citicoline (0.1 µM). After 24 hours, cultures were terminated and analyzed using the MTT assay, AO-EtBr staining, and quantitative reverse transcription-polymerase chain reaction (qRT-PCR). The experimental design can be described as follows.

Analyses of mRNA levels using qRT-PCR

At the end of the experiment (48 hours), the total RNA from the cells was extracted using TRIzol reagent (Sigma-Aldrich, St. Louis, Missouri, United States) according to the manufacturer's procedure. After confirming RNA quality with NanoDrop (Thermo Fisher Scientific, Karlsruhe, Germany), known quantities were reverse-transcribed into cDNA with the Omniscript Kit from Qiagen (Germantown, Maryland, United States). The cDNA was utilized to detect the mRNA levels of many genes related to apoptosis and necrosis. BDNF and neuregulin were also tested using the primer sequences provided in Table 1. Glyceraldehyde-3-phosphate dehydrogenase (GAPDH) was employed as an internal control to standardize the expressions.

**Table 1:** Forward and reverse primer sequences for gene expression studies BDNF: brain-derived neurotrophic factor; GAPDH: glyceraldehyde-3-phosphate dehydrogenase

Gene	Forward primer sequences	Reverse primer sequences
BDNF	5’ CGAGAGGTCTGACGACGACATC 3’	5’ AACCTTCTGGTCCTCATCCAGC 3’
Neuregulin	Forward 5’ CACCCAAGTCAGGAACTCAGCC 3’	Reverse 5’ TGGTCCCAGTCGTGGATGTAGATG 3’
GAPDH	Forward 5’ CGACTTCAACAGCAACTCCCACTC 3’	Reverse 5’ GTAGCCGTATTCATTGTCATACCAGG 3’

Real-time PCR was carried out on a StepOne platform (Applied Biosystems, Waltham, Massachusetts, United States) with Faststart Universal SYBR Green Master Mix (Roche, Indianapolis, Indiana, United States). After the amplification cycles, melt curve analyses were undertaken to rule out any non-specific amplifications. mRNA expression levels were determined using the 2^−ΔΔCT^ technique [13], with the control group as the reference. mRNA expression levels were expressed as the mean of fold changes from three different experiments plus the standard error of the mean. One-way analysis of variance (ANOVA) was used to examine expression level differences at a significance threshold of p<0.05.

## Results

Optimizing the cell culture system

**Figure 1:**
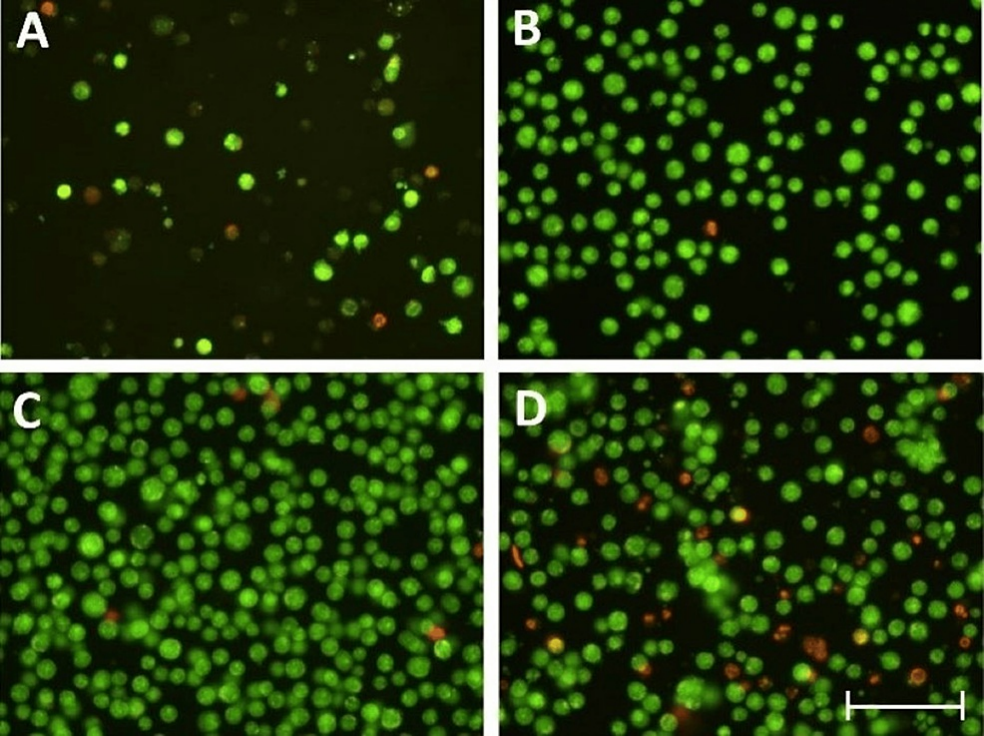
Effect of different serum concentrations (A: 0%, B: 2%, C: 10%, and D: 20%) on Neuro-2A cell population after AO-EtBr staining AO-EtBr: acridine orange/ethidium bromide

Neuro-2A, as an immortalized cell line, tends to divide following a toxic exposure, making cytoprotective tests challenging. To address this issue, different serum supplementation concentrations in the media were evaluated for their proliferative and toxic effects. As expected, no serum supplementation (0%) resulted in cell death. Interestingly, a high serum concentration of 20% was also proven to be harmful. While both 2% and 10% were found to be good, serum cells thrived at 2% but did not increase in quantity much (Figure 1). The results suggest that 2% serum was found to produce a viable and stable population of Neuro-2A, which could be excellent for assessing the neuroprotective properties of the drugs to be evaluated.

Induction of oxidative stress

**Figure 2:**
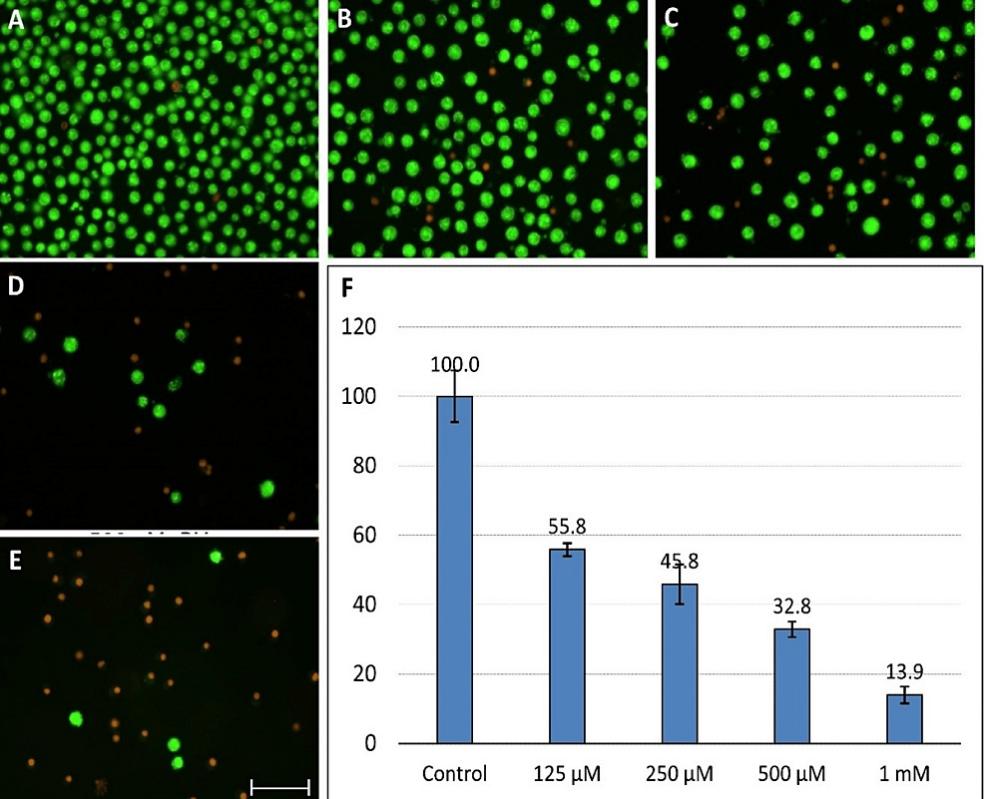
A comparative study of control (A) cells treated with different concentrations of tBuOOH (B: 125 µM, C: 250 µM, D: 500 µM, and E: 1 mM) indicates an increased degree of necrosis with concentration. (F) MTT assay indicates 45.8% of viable healthy cells with 250 µM of tBuOOH tBuOOH: tert-butyl hydroperoxide; MTT: 3-(4,5-dimethylthiazol-2-yl)-2,5-diphenyltetrazolium bromide

Neuro-2A cells that had been cultured in 2% serum for 24 hours were treated with various doses of tBuOOH for 45 minutes before being returned to the original medium. This stimulates the production of oxidative stress and the reperfusion conditions for neurons. After reperfusion, 250 µM of tBuOOH caused approximately 50% cell death (Figure 2).

The optimal dose of citicoline and cerebrolysin

**Figure 3:**
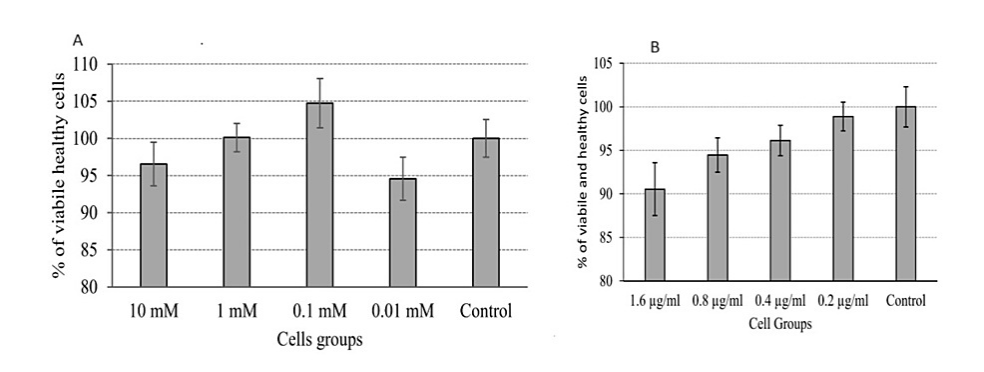
(A) Effect of citicoline on Neuro-2A cells. (B) Effect of cerebrolysin on Neuro-2A cells

Cells cultured with 2% serum for 24 hours were treated with various concentrations of citicoline and cerebrolysin using the same technique utilized to optimize serum concentration and determine the tBuOOH LD50 dosage. Cerebrolysin at 0.2 µg/ml and citicoline at 0.1 µM were determined to have the least cytotoxic effects (Figure 3A, 3B) and considered for further studies.

Neuroprotective effects of citicoline and cerebrolysin

**Figure 4:**
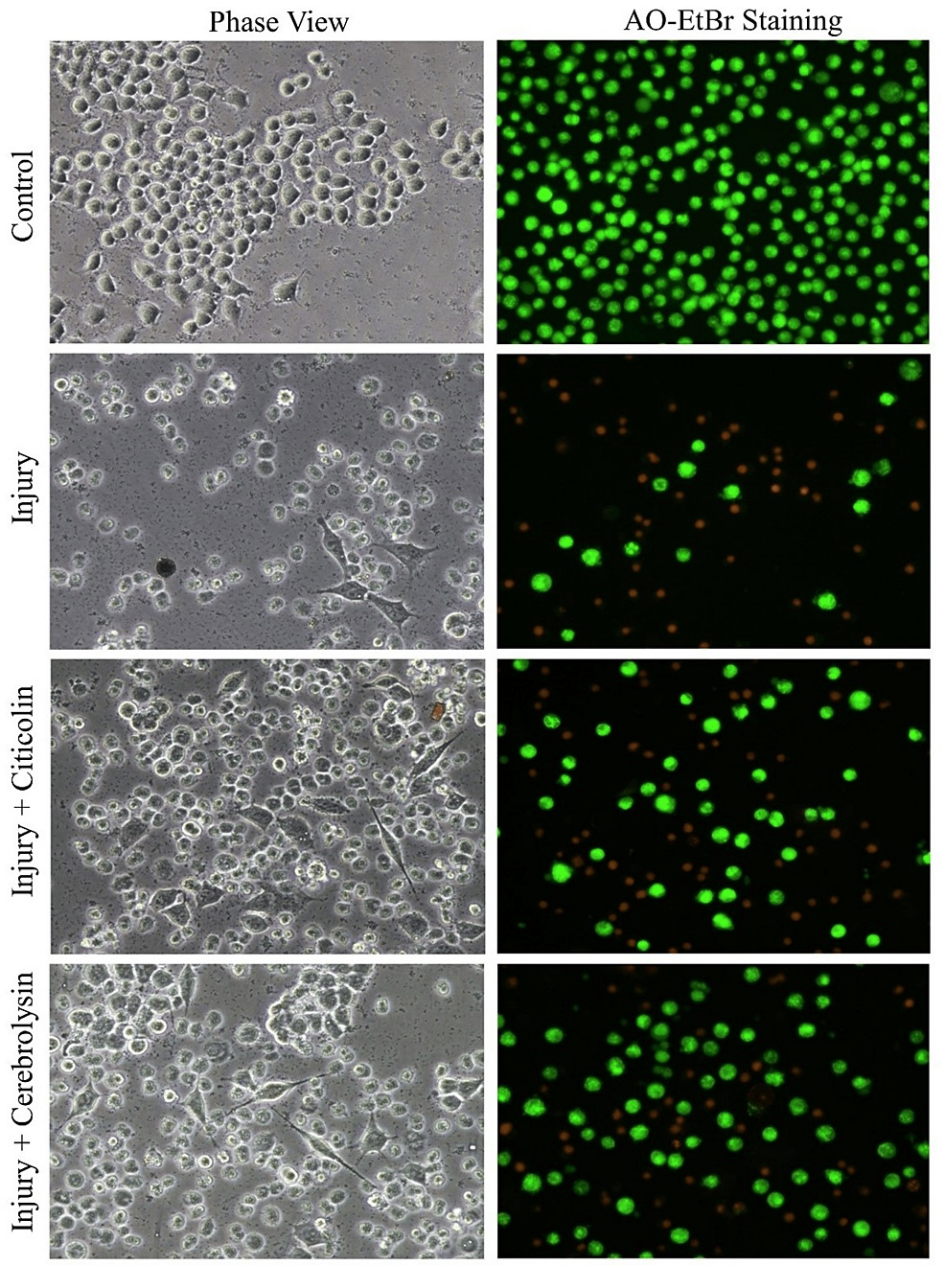
Neuroprotective effect of citicoline and cerebrolysin on Neuro-2A cells subjected to oxidative injury as evident from the increase in cell number and reduction of necrotic cells (orange/red) in AO-EtBr staining. Note: images in phase view and AO-EtBr panels do not represent the same microscopic field AO-EtBr: acridine orange/ethidium bromide

**Figure 5:**
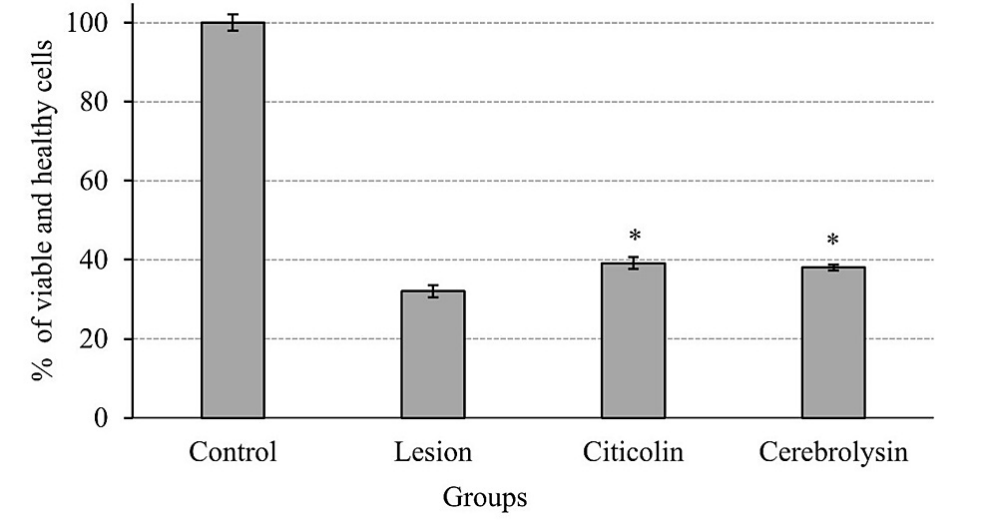
MTT assay shows the cytoprotective effect of citicoline and cerebrolysin on oxidative injury. * represents statistical significance compared with the lesion group at p<0.05. There was no significant difference between citicoline and cerebrolysin in the neuroprotective effect MTT: 3-(4,5-dimethylthiazol-2-yl)-2,5-diphenyltetrazolium bromide

After optimizing serum concentration, tBuOOH dose to cause oxidative stress, and citicoline and cerebrolysin doses, the neuroprotective effects of these drugs were investigated. Following the experimental design described in the Material and Methods section, it was discovered that both citicoline and cerebrolysin improved the survival of Neuro-2A cells after oxidative injury. The difference in neuroprotective impact between citicoline and cerebrolysin was not significant, indicating that their protective effects are nearly identical (Figure 4 and Figure 5).

Gene expression changes induced by citicoline and cerebrolysin

**Figure 6:**
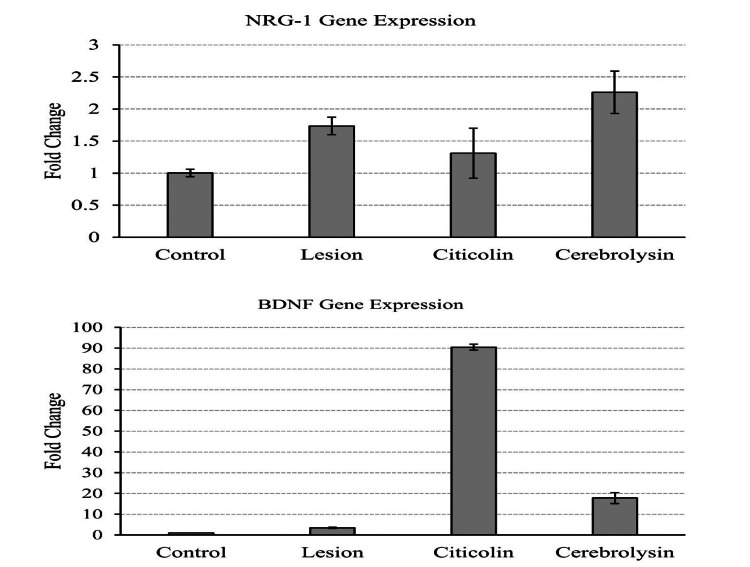
Gene expression changes by citicoline and cerebrolysin. Elevated levels of NRG-1 and BDNF expression are observed as a self-defense response to injury

When compared to the control group, the injury/lesion group showed elevated expressions of Neuregulin 1 (NRG-1) and BDNF. As a result, such an increase could constitute a self-defensive elevation of gene expressions triggered by injury. Cerebrolysin was discovered to significantly boost the expression of both of these genes. Citicoline did not boost NRG-1 expression while significantly increasing BDNF gene expression (Figure 6). Both drugs examined did not significantly alter BCL-2 expression or other cell death-related genes (results not shown).

## Discussion

Kennedy synthesized citicoline (cytidine 5'-diphosphocholine) and described its properties in 1956 [7]. Animal studies have shown that citicoline has beneficial effects on a variety of neurological diseases [14-16]. Shuaib et al. [17] determined that citicoline has neuroprotective properties using an ischemic stroke model in rats. However, clinical research utilizing citicoline produced conflicting outcomes. Alvarez-Sabín and Román [8] argued that citicoline possesses neurogenesis and neurorepair benefits, while Overgaard [9] acknowledged good results but also highlighted issues surrounding its use. Citicoline was shown to be ineffective in a phase III trial, with only a few post hoc studies indicating benefit [18,19]. Surprisingly, citicoline proved ineffective in two major clinical trials [20,21].

Dr. Gerhart Harrer created cerebrolysin in 1949 through the enzymatic hydrolysis of brain tissue, which he claimed had neurotrophic properties. Many studies have been undertaken since then. Cerebrolysin has been shown to have a variety of favorable effects in both basic science investigations using cell cultures [22,23] and animal studies [24]. Eventually, clinical trial results on the efficacy of cerebrolysin began to arrive [25]. Exhaustive recent research found that cerebrolysin has no beneficial impact and may even pose hazards [26]. More importantly, in 2017, an editorial in the journal Stroke [27] found that the use of cerebrolysin for acute stroke therapy is inappropriate and emphasized the need for more randomized trials. The sole consolation was that no major adverse effects were recorded from the usage of cerebrolysin in human patients, despite the likelihood of harm being indicated in an in vitro investigation [28].

From these experiences, it is clear that both citicoline and cerebrolysin may have some benefits, but even after five decades of use, there is still no consensus. Both of these medications have only been approved in a few countries, with the majority of others still awaiting approval. Under these circumstances, it may be preferable to take a fresh look at the problem through basic science studies.

Using the culture technique described here, we were able to maintain a stable population of Neuro-2A cells for 48 hours, providing us with sufficient therapeutic windows of 24 hours to investigate the rescue impact of the drugs utilized post injury. The current study found that both citicoline and cerebrolysin rescued neurons subjected to oxidative stress and that this positive effect was small but statistically significant. Real-time PCR-based mRNA estimate studies provided some insights into the probable mechanism responsible for the observed effect. mRNA levels of genes involved in the cell death pathway, such as Casp3, Bcl2, and others, were unaltered. NRG-1 and BDNF levels were altered, as shown.

NRG-1 serves several different functions in the central nervous system. Aside from its recognized role in myelination [29-34], it was discovered to play a significant role in rescue following ischemia damage such as a stroke [35]. NRG-1 levels were higher in the cerebrolysin group than in the lesion group. BDNF promotes cell survival, differentiation, and synaptic function [36,37]. Alzheimer's disease is associated with BDNF deficiency, which causes neuronal degeneration [38-42]. Its expression has also been shown to be decreased in Parkinson's disease, stress, and anxiety [43,44].

Given the overwhelming support for the role of BDNF in neuronal repair, survival, and degeneration prevention, the observation of significantly upregulated BDNF gene expressions by both citicoline and cerebrolysin could indicate the possible involvement of growth factor-based rescue in oxidatively stressed cells. The lesion group had higher levels of NRG-1 and BDNF than the control group. This could be related to the innate defense mechanisms that these neurons activate in response to the injury. The increased expression of these genes in the treated groups, particularly BDNF levels, may imply that these two medications improve the defense response demonstrated by injured neurons, a hitherto unexplored possibility.

## Conclusions

The effects of citicoline are frequently debated, and no particular mechanism was proposed for cerebrolysin. The present investigation reveals that these two medications have a minor therapeutic effect in rescuing neurons following oxidative stress through the elevation of BDNF gene expression rather than direct engagement in cell death or repair mechanisms. However, complex gene expression studies (combining qRT-PCR assessments of other genes with phospho-specific Western blotting) are needed to confirm this hypothesis.

## References

[1] Xing C, Arai K, Lo EH, Hommel M (2012). Pathophysiologic cascades in ischemic stroke. Int J Stroke.

[2] Segura T, Calleja S, Jordan J (2008). Recommendations and treatment strategies for the management of acute ischemic stroke. Expert Opin Pharmacother.

[3] Prabhakaran S, Ruff I, Bernstein RA (2015). Acute stroke intervention: a systematic review. JAMA.

[4] Windisch M, Gschanes A, Hutter-Paier B (1998). Neurotrophic activities and therapeutic experience with a brain derived peptide preparation. J Neural Transm Suppl.

[5] Chang WH, Park CH, Kim DY (2016). Cerebrolysin combined with rehabilitation promotes motor recovery in patients with severe motor impairment after stroke. BMC Neurol.

[6] Zhang D, Dong Y, Li Y, Chen J, Wang J, Hou L (2017). Efficacy and safety of cerebrolysin for acute ischemic stroke: a meta-analysis of randomized controlled trials. Biomed Res Int.

[7] Kennedy EP (1956). The synthesis of cytidine diphosphate choline, cytidine diphosphate ethanolamine, and related compounds. J Biol Chem.

[8] Alvarez-Sabín J, Román GC (2013). The role of citicoline in neuroprotection and neurorepair in ischemic stroke. Brain Sci.

[9] Overgaard K (2014). The effects of citicoline on acute ischemic stroke: a review. J Stroke Cerebrovasc Dis.

[10] Agarwal S, Patel BM (2017). Is aura around citicoline fading? A systemic review. Indian J Pharmacol.

[11] Mironova EV, Evstratova AA, Antonov SM (2007). A fluorescence vital assay for the recognition and quantification of excitotoxic cell death by necrosis and apoptosis using confocal microscopy on neurons in culture. J Neurosci Methods.

[12] Muthaiah VP, Michael FM, Palaniappan T, Rajan SS, Chandrasekar K, Venkatachalam S (2017). JNK1 and JNK3 play a significant role in both neuronal apoptosis and necrosis. Evaluation based on in vitro approach using tert-butylhydroperoxide induced oxidative stress in neuro-2A cells and perturbation through 3-aminobenzamide. Toxicol In Vitro.

[13] Schmittgen TD, Livak KJ (2008). Analyzing real-time PCR data by the comparative C(T) method. Nat Protoc.

[14] Dixon CE, Ma X, Marion DW (1997). Effects of CDP-choline treatment on neurobehavioral deficits after TBI and on hippocampal and neocortical acetylcholine release. J Neurotrauma.

[15] Fresta M, Puglisi G (1997). Survival rate improvement in a rat ischemia model by long circulating liposomes containing cytidine-5I-diphosphate choline. Life Sci.

[16] Bruhwyler J, Liégeois JF, Géczy J (1998). Facilitatory effects of chronically administered citicoline on learning and memory processes in the dog. Prog Neuropsychopharmacol Biol Psychiatry.

[17] Shuaib A, Yang Y, Li Q (2000). Evaluating the efficacy of citicoline in embolic ischemic stroke in rats: neuroprotective effects when used alone or in combination with urokinase. Exp Neurol.

[18] Clark WM, Wechsler LR, Sabounjian LA, Schwiderski UE (2001). A phase III randomized efficacy trial of 2000 mg citicoline in acute ischemic stroke patients. Neurology.

[19] Ali Mousavi S, Khorvash F, Hoseini T (2010). The efficacy of citroline in the treatment of ischemic stroke and primary hypertensive intracereral hemorrhage; a review article. ARYA Atheroscler.

[20] Dávalos A, Alvarez-Sabín J, Castillo J (2012). Citicoline in the treatment of acute ischaemic stroke: an international, randomised, multicentre, placebo-controlled study (ICTUS trial). Lancet.

[21] Zafonte RD, Bagiella E, Ansel BM (2012). Effect of citicoline on functional and cognitive status among patients with traumatic brain injury: Citicoline Brain Injury Treatment Trial (COBRIT). JAMA.

[22] Lombardi VR, Windisch M, García M, Cacabelos R (1999). Effects of cerebrolysin on in vitro primary microglial and astrocyte rat cell cultures. Methods Find Exp Clin Pharmacol.

[23] Hartbauer M, Hutter-Paie B, Windisch M (2001). Effects of cerebrolysin on the outgrowth and protection of processes of cultured brain neurons. J Neural Transm (Vienna).

[24] Zhang L, Chopp M, Lu M (2016). Cerebrolysin dose-dependently improves neurological outcome in rats after acute stroke: a prospective, randomized, blinded, and placebo-controlled study. Int J Stroke.

[25] Muresanu DF, Heiss WD, Hoemberg V (2016). Cerebrolysin and Recovery After Stroke (CARS): a randomized, placebo-controlled, double-blind, multicenter trial. Stroke.

[26] Ziganshina LE, Abakumova T, Hoyle CH (2020). Cerebrolysin for acute ischaemic stroke. Cochrane Database Syst Rev.

[27] Bereczki D (2017). Hope dies last-evidence again fails to support a neuroprotectant: cerebrolysin for acute ischemic stroke. Stroke.

[28] Keilhoff G, Lucas B, Pinkernelle J, Steiner M, Fansa H (2014). Effects of cerebrolysin on motor-neuron-like NSC-34 cells. Exp Cell Res.

[29] Brinkmann BG, Agarwal A, Sereda MW (2008). Neuregulin-1/ErbB signaling serves distinct functions in myelination of the peripheral and central nervous system. Neuron.

[30] Snaidero N, Simons M (2017). The logistics of myelin biogenesis in the central nervous system. Glia.

[31] Makinodan M, Rosen KM, Ito S, Corfas G (2012). A critical period for social experience-dependent oligodendrocyte maturation and myelination. Science.

[32] Lundgaard I, Luzhynskaya A, Stockley JH (2013). Neuregulin and BDNF induce a switch to NMDA receptor-dependent myelination by oligodendrocytes. PLoS Biol.

[33] Zhang JF, Zhao FS, Wu G (2011). Therapeutic effect of co-transplantation of neuregulin-1-transfected Schwann cells and bone marrow stromal cells on spinal cord hemisection syndrome. Neurosci Lett.

[34] Gauthier MK, Kosciuczyk K, Tapley L, Karimi-Abdolrezaee S (2013). Dysregulation of the neuregulin-1-ErbB network modulates endogenous oligodendrocyte differentiation and preservation after spinal cord injury. Eur J Neurosci.

[35] Xu Z, Croslan DR, Harris AE, Ford GD, Ford BD (2006). Extended therapeutic window and functional recovery after intraarterial administration of neuregulin-1 after focal ischemic stroke. J Cereb Blood Flow Metab.

[36] Kaplan DR, Miller FD (2000). Neurotrophin signal transduction in the nervous system. Curr Opin Neurobiol.

[37] Chao MV (2003). Neurotrophins and their receptors: a convergence point for many signalling pathways. Nat Rev Neurosci.

[38] Li N, Liu GT (2010). The novel squamosamide derivative FLZ enhances BDNF/TrkB/CREB signaling and inhibits neuronal apoptosis in APP/PS1 mice. Acta Pharmacol Sin.

[39] Counts SE, Mufson EJ (2005). The role of nerve growth factor receptors in cholinergic basal forebrain degeneration in prodromal Alzheimer disease. J Neuropathol Exp Neurol.

[40] Fumagalli F, Racagni G, Riva MA (2006). The expanding role of BDNF: a therapeutic target for Alzheimer's disease?. Pharmacogenomics J.

[41] Phillips HS, Hains JM, Armanini M, Laramee GR, Johnson SA, Winslow JW (1991). BDNF mRNA is decreased in the hippocampus of individuals with Alzheimer's disease. Neuron.

[42] Tapia-Arancibia L, Aliaga E, Silhol M, Arancibia S (2008). New insights into brain BDNF function in normal aging and Alzheimer disease. Brain Res Rev.

[43] Fumagalli F, Racagni G, Riva MA (2006). Shedding light into the role of BDNF in the pharmacotherapy of Parkinson's disease. Pharmacogenomics J.

[44] Martinowich K, Manji H, Lu B (2007). New insights into BDNF function in depression and anxiety. Nat Neurosci.

